# Knockdown of circ_0005615 enhances the radiosensitivity of colorectal cancer by regulating the miR-665/NOTCH1 axis

**DOI:** 10.1515/med-2023-0678

**Published:** 2023-05-09

**Authors:** Ximin Wang, Dongxu Zheng, Changting Wang, Wanhua Chen

**Affiliations:** Department of Clinical Laboratory, The Second Affiliated Hospital of Fujian Medical University, Licheng, Quanzhou, Fujian, 362000, P. R. China; Department of General Surgery, The Second Affiliated Hospital of Fujian Medical University, Licheng, Quanzhou, Fujian, 362000, P. R. China; Department of Clinical Laboratory, The Second Affiliated Hospital of Fujian Medical University, 34 Zhongshan North Road, Licheng, Quanzhou, Fujian, 362000, P. R. China

**Keywords:** circular RNAs, circ_0005615, miR-665, NOTCH1, colorectal cancer, radiosensitivity

## Abstract

Radiotherapy resistance is a challenge for colorectal cancer (CRC) treatment. Circular RNAs (circRNAs) play vital roles in the occurrence and development of CRC. This study aimed to investigate the role of circ_0005615 in regulating the radiosensitivity of CRC. The levels of circ_0005615, microRNA-665 (miR-665), and notch receptor 1 (NOTCH1) were detected by quantitative real-time PCR or western blot. The radiosensitivity of CRC cells was assessed by colony formation assay. Cell viability, apoptosis, and colony formation were assessed by Cell Counting Kit-8 assay, flow cytometry, and colony formation assay. Cell migration and invasion were confirmed by transwell assay and scratch assay. The binding relationship between miR-665 and circ_0005615 or NOTCH1 was verified by dual-luciferase reporter assay. Xenograft assay was used to test the effect of circ_0005615 on radiosensitivity *in vivo*. circ_0005615 and NOTCH1 were up-regulated, and miR-665 was down-regulated in CRC tissues and cells. Radiation decreased circ_0005615 and NOTCH1 levels and increased miR-665 level. Knockdown of circ_0005615 enhanced radiosensitivity of CRC cells. Moreover, circ_0005615 sponged miR-665 to regulate the radioresistance of CRC cells. Besides, miR-665 targeted NOTCH1 to mediate the radiosensitivity of CRC cells. Furthermore, circ_0005615 depletion increased CRC radiosensitivity *in vivo*. circ_0005615 silencing elevated radiosensitivity of CRC by regulating miR-665/NOTCH1 axis.

## 1 Introduction

Colorectal cancer (CRC) is a common malignancy, ranking third in cancer incidence and second in mortality [1]. Unfortunately, CRC is usually asymptomatic in the early stage, and diagnosis often occurs in the advanced stage [2]. Surgery combined with adjuvant chemotherapy and radiotherapy is the preferred choice for CRC patients [3]. Additionally, a combination of radiotherapy and chemotherapy is considered a common treatment for locally advanced CRC [4]. Nevertheless, acquired or inherent drug resistance and radioresistance lead to limited therapeutic effects [5]. Therefore, revealing the molecular regulation of drug resistance and radioresistance is crucial for CRC treatment.

Circular RNAs (circRNAs) are single-stranded molecules with covalent closed-loop structures and no 5′ to 3′ polarity [6,7]. Mounting evidence has elucidated that circRNAs are abnormally expressed in cancer and are strongly related to tumor progression [8]. Besides, numerous investigations have highlighted that circRNA dysregulation exerts a crucial regulatory effect on the radiosensitivity of solid tumor cells [9–11]. For instance, circRNA_0000285 was prominently up-regulated in patients with radioresistant nasopharyngeal carcinoma, indicating that circRNA_0000285 might participate in radiosensitivity [12]. In addition, circ_VCAN in cancer cells was significantly down-regulated after irradiation, and circ_VCAN accelerated glioma progression and decreased radiosensitivity of glioma via combining with microRNA-1183 [13]. Moreover, circRNA_014511 facilitated carcinogenesis and reduced the radiosensitivity of bone marrow mesenchymal stem cells via absorbing microRNA-29b-2-5p [14]. Besides, interference of circ_0007031 suppressed tumor cell growth and reduced chemoradiotherapy resistance in CRC [15]. A recent research revealed that circ_0005615 derived from the nuclear factor of activated T cells 3 was highly expressed in CRC, and circ_0005615 silencing impeded cell proliferation and accelerated cell cycle arrest [16]. However, the role of circ_0005615 in radiotherapy has not been studied.

Furthermore, mounting evidence has demonstrated that circRNAs competitively bind to microRNAs (miRNAs), thereby indirectly regulating mRNA expression [17]. Additionally, substantial studies have suggested that some miRNAs can serve as detection factors for the radiosensitivity of cancer [18]. For example, miR-122-5p targeted CCAR1 to accelerate radiation-induced rectal injury and functioned as a radiosensitizer [19]. Chen et al. unveiled that miR-450a-5p restrained autophagy and elevated radiosensitivity in esophageal squamous cell carcinoma through inhibition of DUSP10 [20]. Also, Huang et al. discovered that miR-181a enhanced the radioresistance of nasopharyngeal carcinoma via negatively regulating RKIP [21]. Previous research showed that miR-665 was strikingly down-regulated in CRC [22]. Nonetheless, the function of miR-665 in CRC radiotherapy remains unknown. Apart from that, previous research indicated that notch receptor 1 (NOTCH1) is an oncogene and is corrected with radio-resistance in various cancers [23,24]. Herein, according to bioinformatics analysis, miR-665 was found to possess some binding sites with circ_0005615 or NOTCH1. Therefore, we hypothesized that the regulatory role of circ_0005615 on radio-resistance in CRC might be mediated by the miR-665/NOTCH1 axis.

## 2 Materials and methods

### 2.1 Clinical specimens

Thirty CRC tissues and matched adjacent normal tissues were collected from 30 CRC patients, containing 14 patients with primary CRC (the less-radiosensitive group) and 16 with recurrent CRC (the high-radiosensitive group) after radiation treatment, recruited at the Second Affiliated Hospital of Fujian Medical University. All tissue samples were stored at −80°C immediately after resection. The clinicopathological parameters of CRC patients are presented in Table 1.

**Table 1:** Correlation between circ_0005615 expression and clinicopathological parameters of CRC patients

Parameter	Case	circ_0005615 expression		*P* value^a^
		Low (*n* = 15)	High (*n* = 15)	
Age (years)				0.273
≤50	15	9	6	
>50	15	6	9	
Gender				0.269
Female	13	8	5	
Male	17	7	10	
Tumor size				0.001*
≤5 cm	15	12	3	
>5 cm	15	3	12	
TNM stage				0.003*
I–II	18	13	5	
III	12	2	10	
Lymphatic metastasis				0.003*
Negative	16	12	4	
Positive	14	3	11	

Note: *P < 0.05, ^a^Chi-square test.


**Ethical approval:** All participants carefully read and signed the written informed consent. This research was ratified by the Ethics Committee of the Second Affiliated Hospital of Fujian Medical University. The research has been carried out in accordance with the World Medical Association Declaration of Helsinki, and all subjects provided written informed consent.

### 2.2 Cell culture and radiation treatment

CRC cell lines (LOVO and SW480) were purchased from the American Type Culture Collection (cat. nos. CCL-229 and CCL-228; ATCC, Manassas, VA, USA). Normal colonic epithelial cells (NCM460) were bought from Fuyuanbio (Shanghai, China). All cells were cultured in RPMI-1640 medium (Hyclone, Logan, UT, USA) supplemented with 10% fetal bovine serum (FBS; Hyclone) and maintained in an incubator with 5% CO_2_ at 37°C.

To maintain the radioresistance phenotype, LOVO and SW480 cells were exposed to different doses of 6 MV X-ray (0, 2, 4, 6 and 8 Gy) using a linear accelerator (Varian, Palo Alto, CA, USA). Also, the resistant cells were cultured in a medium containing 6 Gy 6 MV X-ray. After 24 h of irradiation, RNA was collected and used in subsequent experiments.

### 2.3 Cell transfection

circ_0005615 small interfering RNA (si-circ_0005615; 5′-ATTTCGATCTTGAGCCAGA-3′) and the control (si-NC; 5′-CGTTATTACTTGAGCCAGA-3′), miR-665 mimic and negative control (miRNA NC), miR-665 inhibitor and negative control (inhibitor NC), NOTCH1 overexpression vector (pc-NOTCH1) and the control (pc-NC) were purchased from Genechem (Shanghai, China). The oligonucleotides or vectors were introduced into LOVO and SW480 cells at 60% confluency using Lipofectamine 3000 (Invitrogen, Carlsbad, CA, USA).

### 2.4 Quantitative real-time PCR (qRT-PCR)

RNA isolation was performed using Trizol reagent (Solarbio, Beijing, China). Then, the complementary DNA (cDNA) was synthesized using the specific reverse transcription kit (Vazyme, Nanjing, China). Subsequently, the RNA level was examined via AceQ qPCR SYBR Green Master Mix (Vazyme) and quantified via the 2^−ΔΔCt^ method. Glyceraldehyde 3-phosphate dehydrogenase (GAPDH) acted as the internal reference to normalize the expression of circ_0005615 and NOTCH1, and U6 was used as the endogenous control to normalize the expression of miR-665. All primers were shown: circ_0005615-F: 5′-TCACCCTTTACCTGGAGCAAA-3′, circ_0005615-R: 5′-GAGCTGAAACGATGGTGACAAA-3′; miR-665-F: 5′-ACCAGGAGGCTGAGGC-3′, miR-665-R: 5′-GAACATGTCTGCGTATCTC-3′; NOTCH1-F: 5′-GAGGCGTGGCAGACTATGC-3′, NOTCH1-R: 5′-CTTGTACTCCGTCAGCGTGA-3′; GAPDH-F: 5′-GAAGGTGAAGGTCGGAGT-3′, GAPDH-R: 5′-GATGGCAACAATATCCACTT-3′; and U6-F: 5′-CTCGCTTCGGCAGCACA-3′, U6-R: 5′-ACGCTTCACGAATTTGCGT-3′.

### 2.5 Nuclear and cytoplasmic fraction assay

The subcellular localization of circ_0005615 was evaluated using mirVana^TM^ PARIS Kit (Invitrogen). GAPDH and U6 were regarded as positive controls for cytoplasmic and nuclear fractions, respectively.

### 2.6 Colony formation assay

Treated LOVO and SW480 cells were maintained in six-well plates and cultured for 48 h. Subsequently, the cells were treated with various doses of irradiation (0, 2, 4, 6, and 8 Gy). Then, the cells were incubated for 2 weeks at 37°C. After staining with 1% crystal violet (Beyotime, Shanghai, China), the colonies were counted under a microscope.

### 2.7 Cell viability assay

To detect cell viability, Cell Counting Kit-8 (CCK-8) kit (Beyotime) was used in this study. Treated LOVO and SW480 cells (2.0 × 10^3^) were injected into 96-well plates. After incubation at 37°C for 24 h, the cells were reacted with 10 µL CCK-8 solution (Beyotime) for 4 h. Then, cell viability was evaluated by measuring optical density using a Microplate Reader (BioTek, Burlington, VT, USA).

### 2.8 Flow cytometry

Cell apoptosis was monitored using Annexin V-FITC/propidium iodide (PI) Apoptosis Detection kit (Vazyme) according to the manufacturer’s instructions. After LOVO and SW480 cells were treated with 6 Gy irradiation alone or combined with transfection, the cells were stained with Annexin V-FITC and PI. Subsequently, the apoptosis rate was tested by FACScan Flow Cytometer (BD Biosciences, San Diego, CA, USA).

### 2.9 Transwell assay

Cell migration and invasion were assessed using 24-transwell plates with 8 μm pore inserts (Corning, Corning, NY, USA) and transwell chambers with Matrigel (Corning)-precoated inserts, respectively. In short, treated LOVO and SW480 cells were seeded into the upper chamber, and the lower chamber was filled with RPMI-1640 medium containing 10% FBS (Hyclone). After incubation for 24 h, the cells migrated/invaded into the lower surface of the membranes were fixed with methanol and stained with 0.5% crystal violet (Beyotime). Then, the stained cells were counted under a microscope at 100× magnification.

### 2.10 Scratch assay

To check tumor cell migration ability, treated LOVO and SW480 cells (5 × 10^5^) were plated in six-well plates. Subsequently, the cells were scratched with a sterilized pipette tip and the floating cells were removed with PBS (Hyclone). After culturing for 0 or 24 h, the images were taken under a microscope at 100× magnification, and the migrated distance was calculated using ImageJ 1.8.0 software (National Institutes of Health, Bethesda, MD, USA). The migration rate was calculated using the following formula: Migration rate (%) = (S0 h – S24 h)/S0 h × 100. S0 h represents the distance of the scratch at 0 h and S24 h represents the distance at 24 h.

### 2.11 Dual-luciferase reporter assay

To verify the interaction between miR-665 and circ_0005615 or NOTCH1, dual-luciferase reporter assay was conducted. The sequences of circ_0005615 or NOTCH1 3′-UTR harboring miR-665 wild-type or mutant binding sites were cloned into the pmirGLO vector (LMAI Bio, Shanghai, China) to form WT-circ_0005615, MUT-circ_0005615, WT-NOTCH1-3′-UTR, or MUT-NOTCH1-3′-UTR, respectively. Then, the luciferase reporter and miRNA NC or miR-665 mimic were introduced into LOVO and SW480 cells. The luciferase intensity was determined via Dual-Lucy Assay Kit (Solarbio).

### 2.12 Western blot assay

Protein was extracted using RIPA buffer (Solarbio). Then, equal amounts of protein samples were separated by polyacrylamide gel electrophoresis and transferred onto polyvinylidene fluoride membranes (Millipore, Billerica, MA, USA). After blocking with 5% skimmed milk for 2 h, the membranes were probed with primary antibodies against NOTCH1 (ab52627, 1:1,500; Abcam, Cambridge, UK) or GAPDH (ab9485, 1:2,500; Abcam) at 4°C overnight. Afterward, the membranes interacted with a secondary antibody (ab205718, 1:20,000; Abcam) for 2 h at room temperature. Finally, the signal intensity was measured by ECL reagent (Millipore).

### 2.13 Xenograft assay

The experiment was approved by the Animal Ethics Committee of the Second Affiliated Hospital of Fujian Medical University. LOVO cells (5 × 10^6^) stably transfected with sh-NC or sh-circ_0005615 (Genechem) were subcutaneously injected into the right back of BALB/c nude mice (5-week-old; *n* = 10). Nude mice were divided into two groups. One week after injection, the mice received 6 Gy irradiation every 2 days. Tumor volume was monitored once a week. Four weeks later, the mice were sacrificed and the xenograft tumors were weighed. In addition, the levels of circ_0005615, miR-665, and NOTCH1 in xenograft tissues were measured using qRT-PCR and western blot. Animal studies were performed in compliance with the ARRIVE guidelines and the Basel Declaration. All animals received humane care according to the National Institutes of Health (USA) guidelines.

### 2.14 Statistical analysis

All data were displayed as mean ± standard deviation (SD) in three independent replicates by using Graphpad Prism 7.0 software (GraphPad, San Diego, CA, USA). Student’s *t*-test and one-way analysis of variance were utilized to assess statistical differences. *P* < 0.05 was considered statistically significant.

## 3 Results

### 3.1 circ_0005615 is up-regulated in CRC tissues and cells

**Figure 1:**
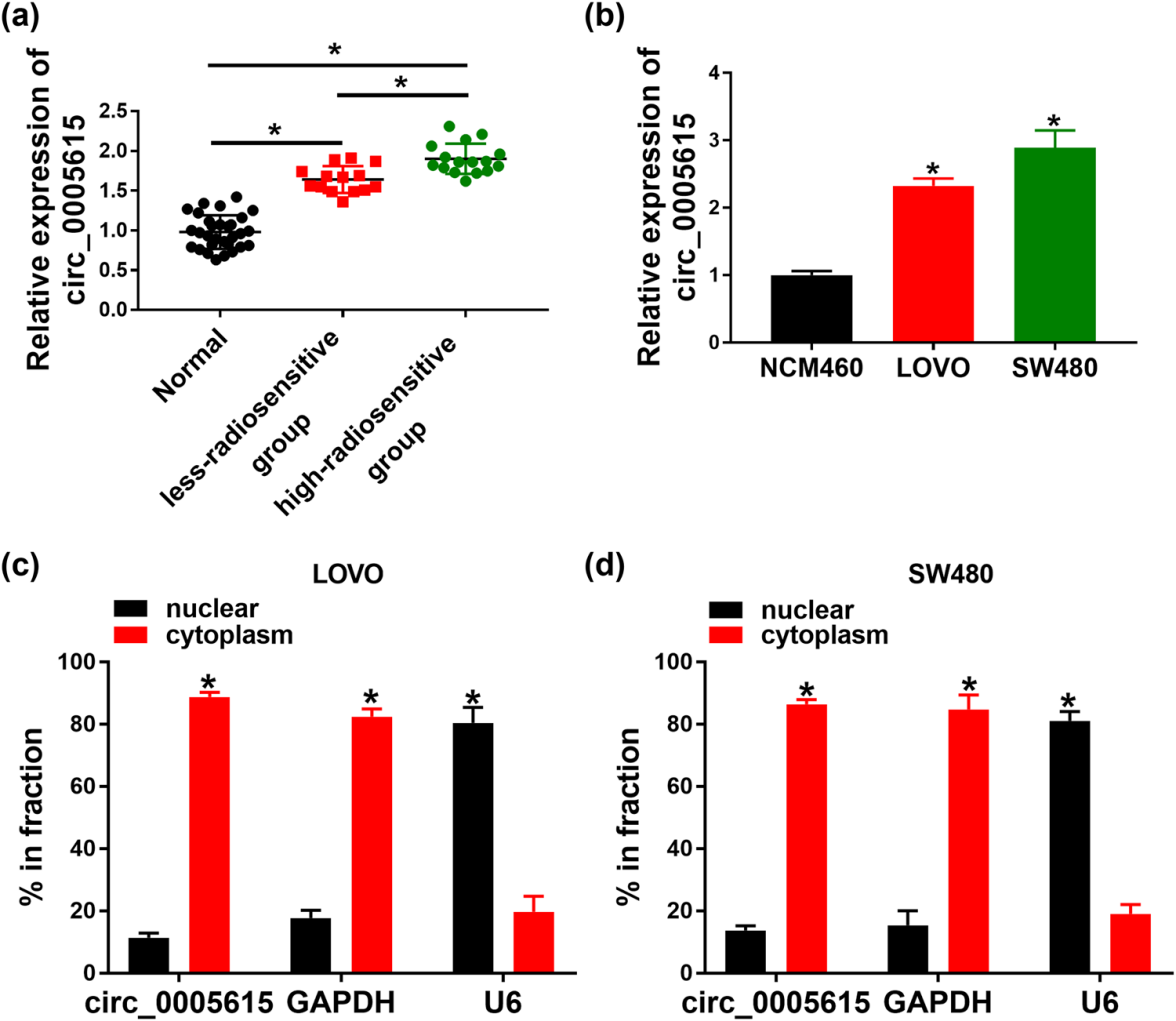
circ_0005615 is up-regulated in CRC tissues and cells. (a) circ_0005615 expression in normal tissues (*n* = 30), the less-radiosensitive group (*n* = 14), and the high-radiosensitive group (*n* = 16) was examined by qRT-PCR. (b) The level of circ_0005615 was measured in NCM460, LOVO, and SW480 cells (*n* = 3). (c and d) Levels of circ_0005615, GAPDH, and U6 were detected in nuclear and cytoplasmic fractions (*n* = 3). Data are shown as mean ± SD. *P < 0.05.

To explore the role of circ_0005615 in CRC, we first determined the expression of circ_0005615 in CRC tissues and normal tissues. As depicted in Figure 1a, circ_0005615 level was increased in CRC tissues compared with normal tissues. In order to check the function of circ_0005615 in radioresistance, its expression was measured in the less-radiosensitive group (*n* = 14) or the high-radiosensitive group (*n* = 16). Compared with the less-radiosensitive group, circ_0005615 level was apparently enhanced in the high-radiosensitive group (Figure 1a). Similarly, circ_0005615 expression was strikingly higher in LOVO (2.32-fold) and SW480 cells (2.98-fold) than that in NCM460 cells (Figure 1b). In addition, nuclear and cytoplasmic fraction assay suggested that circ_0005615 was mainly distributed in the cytoplasm (Figure 1c and d). As shown in Table 1, circ_0005615 expression was not associated with age and gender but was related to tumor size, TNM stage, and lymphatic metastasis. These data hinted that circ_0005615 might play an oncogene role in CRC.

### 3.2 Depletion of circ_0005615 enhances the radiosensitivity of CRC cells

**Figure 2:**
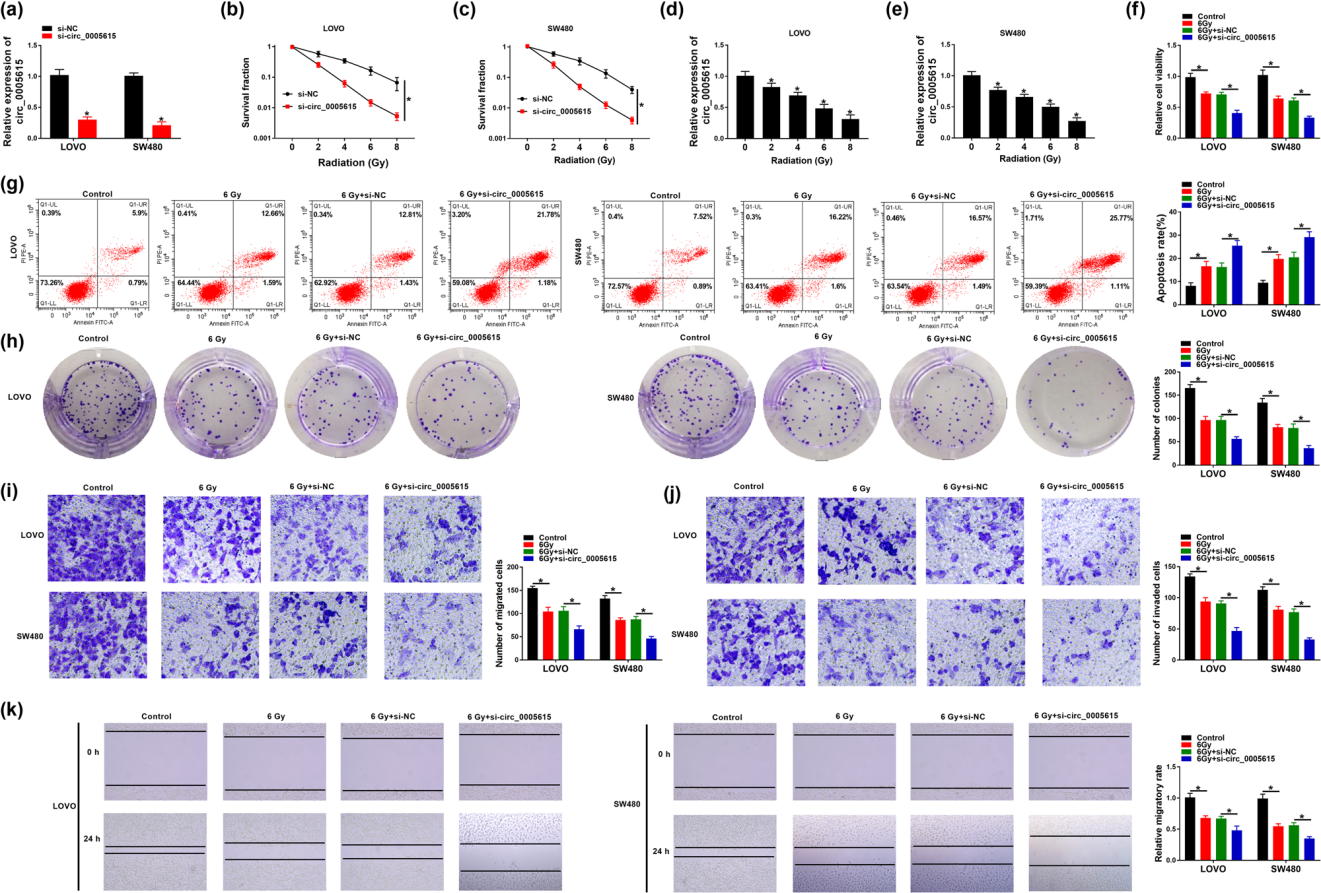
Depletion of circ_0005615 enhances the radiosensitivity of CRC cells. (a) Transfection efficiency of si-circ_0005615 in LOVO and SW480 cells was determined by qRT-PCR. (b and c) Colony formation assay was performed in LOVO and SW480 cells transfected with si-NC or si-circ_0005615 exposed to different doses of X-ray. (d and e) Expression of circ_0005615 was examined in LOVO and SW480 cells treated with various doses of X-ray. After LOVO and SW480 cells were exposed to 6 Gy irradiation alone or in combination with si-circ_0005615, cell viability (f), apoptosis (g), colony number (h), migration and invasion (i and j), and migratory rate (k) were detected by CCK-8 assay, flow cytometry, colony formation assay, transwell, and scratch assay. Data are shown as mean ± SD, *n* = 3. *P < 0.05.

To illuminate the function of circ_0005615 in radiosensitivity, loss-of-function experiments were carried out in LOVO and SW480 cells transfected with si-NC or si-circ_0005615. First, qRT-PCR confirmed that the knockdown efficiency of circ_0005615 was significant in LOVO (with a 70% reduction) and SW480 (with a 79% reduction) cells (Figure 2a). Moreover, colony formation assay showed that circ_0005615-silenced CRC cells were more sensitive to X-ray irradiation (Figure 2b and c). Meanwhile, radiation reduced the expression of circ_0005615 in a dose-dependent manner (Figure 2d and e). Next, LOVO and SW480 cells were treated with 6 Gy irradiation alone or together with si-circ_0005615. CCK-8 analysis showed that radiation treatment remarkably reduced the viability of LOVO and SW480 cells, and si-circ_0005615 transfection enhanced this effect (Figure 2f). Flow cytometry suggested that circ_0005615 knockdown strengthened the promotion of cell apoptosis caused by radiation alone (Figure 2g). Colony formation assay exhibited that radiation significantly decreased the number of colonies in LOVO and SW480 cells, and this change was enhanced after the introduction of si-circ_0005615 (Figure 2h). Besides, transwell and scratch assays showed that circ_0005615 silence aggravated the inhibitory effect of radiation on cell migration and invasion (Figure 2i–k). Collectively, these data evidenced that circ_0005615 knockdown increased the radiosensitivity of CRC cells.

### 3.3 circ_0005615 directly interacts with miR-665

**Figure 3:**
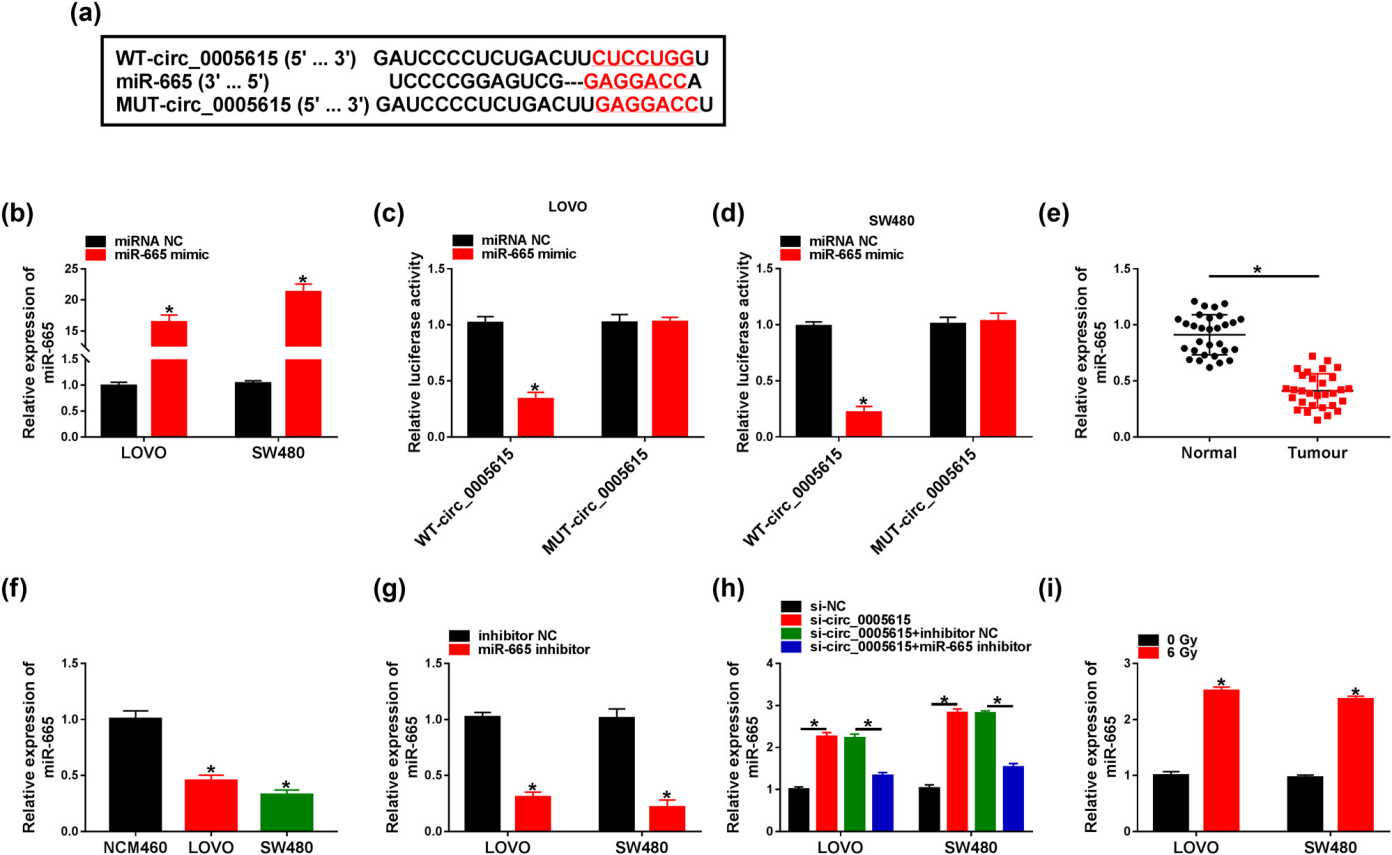
circ_0005615 directly interacts with miR-665. (a) Putative binding site of miR-665 in circ_0005615 is shown. (b) Overexpression efficiency of miR-665 was detected using qRT-PCR (*n* = 3). (c and d) Luciferase activity was tested in LOVO and SW480 cells co-transfected with WT-circ_0005615 or MUT-circ_0005615 and miRNA NC or miR-665 mimic (*n* = 3). (e) miR-665 level was determined in CRC tissues and adjacent normal tissues (*n* = 30). (f) miR-665 level was examined in NCM460, LOVO, and SW480 cells (*n* = 3). (g) Transfection efficiency of miR-665 inhibitor was detected by qRT-PCR (*n* = 3). (h) miR-665 level was measured in LOVO and SW480 cells transduced with si-NC, si-circ_0005615, si-circ_0005615 + inhibitor NC, or si-circ_0005615 + miR-665 inhibitor (*n* = 3). (i) Expression of miR-665 was detected in LOVO and SW480 cells treated with 6 Gy irradiation (*n* = 3). Data are shown as mean ± SD. *P < 0.05.

The online database circinteractome predicted that circ_0005615 contained a possible miR-665 binding site (Figure 3a). Subsequently, the miR-665 level was prominently increased in LOVO (16.39-fold) and SW480 (21.25-fold) cells transfected with miR-665 mimic compared to the control group (Figure 3b). Dual-luciferase reporter assay was performed to verify the relationship between circ_0005615 and miR-665, and the results showed that miR-665 mimic overtly reduced the luciferase activity of WT-circ_0005615 reporter (Figure 3c and d). Compared with the control group, miR-665 expression was strikingly decreased in CRC tissues (with a 65% reduction) and cells (LOVO cells with a 55% decrease, SW480 cells with a 67% reduction) (Figure 3e and f). Next, the knockdown efficiency of miR-665 was determined by transfecting miR-665 inhibitor into LOVO (with a 70% decrease) and SW480 cells (with an 80% reduction) (Figure 3g). Furthermore, silencing of circ_0005615 remarkably increased miR-665 level, while the change was abrogated after transfection with the miR-665 inhibitor (Figure 3h). In addition, 6 Gy irradiation elevated miR-665 expression in LOVO (2.51-fold) and SW480 (2.36-fold) cells (Figure 3i).

### 3.4 circ_0005615 affects the radiosensitivity of CRC cells via regulating miR-665

**Figure 4:**
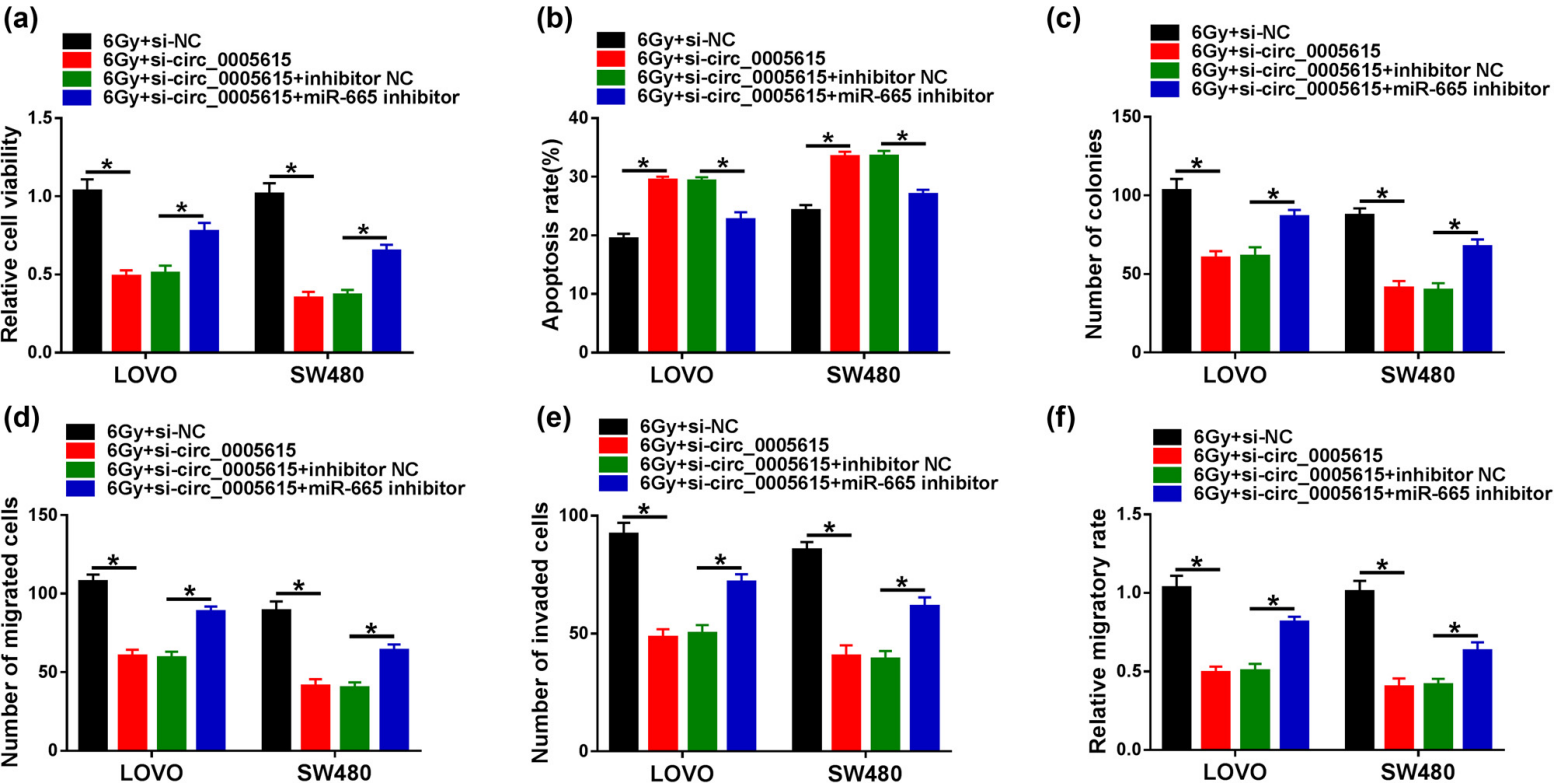
circ_0005615 affects the radiosensitivity of CRC cells via regulating miR-665. LOVO and SW480 cells were transfected with si-NC, si-circ_0005615, si-circ_0005615 + inhibitor NC, or si-circ_0005615 + miR-665 inhibitor and then exposed to 6 Gy irradiation. Cell viability (a), apoptosis (b), colony number (c), migration and invasion (d and e), and migratory rate (f) were examined using CCK-8 assay, flow cytometry, colony formation assay, transwell, and scratch assay, respectively. Data are shown as mean ± SD, *n* = 3. *P < 0.05.

To investigate whether circ_0005615 targeted miR-665 to modulate the radiosensitivity of CRC cells, LOVO and SW480 cells were introduced with si-NC, si-circ_0005615, si-circ_0005615 + inhibitor NC, or si-circ_0005615 + miR-665 inhibitor, followed by 6 Gy radiation treatment. CCK-8 and flow cytometry assays exhibited that circ_0005615 knockdown inhibited cell viability and induced apoptosis in LOVO and SW480 cells, whereas these effects were partially alleviated by down-regulating miR-665 (Figure 4a and b). Moreover, transfection with miR-665 inhibitor partially attenuated the reduction in colony number caused by circ_0005615 silencing (Figure 4c). Additionally, down-regulation of circ_0005615 impeded the migration and invasion of LOVO and SW480 cells, while these impacts were overturned by inhibiting miR-665 (Figure 4d–f). Overall, these data indicated that circ_0005615 depletion increased the radiosensitivity of CRC cells by modulating miR-665.

### 3.5 NOTCH1 is a target of miR-665

**Figure 5:**
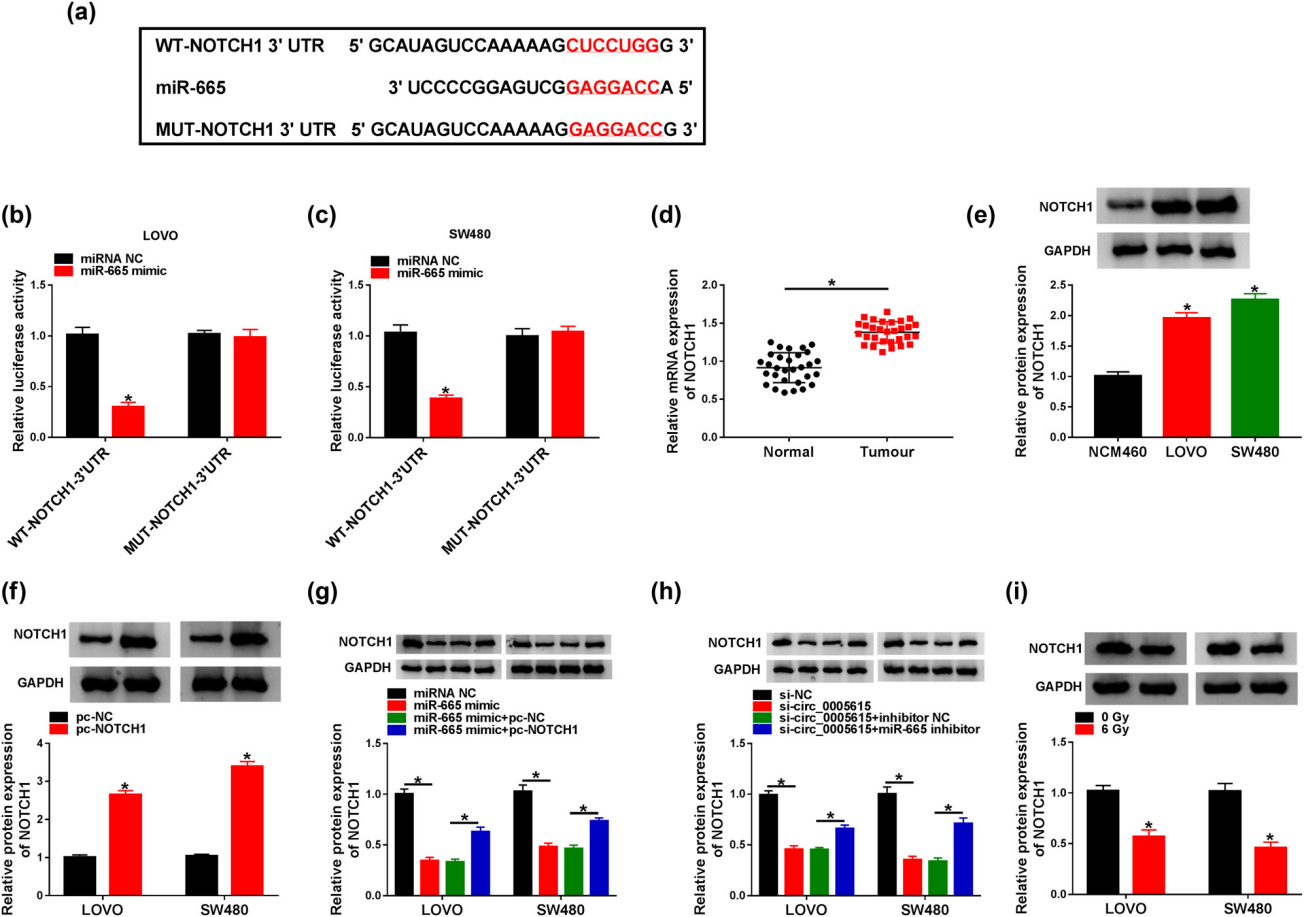
NOTCH1 is a target of miR-665. (a) Putative binding site of miR-665 in NOTCH1 3′-UTR was displayed. (b and c) Luciferase activity was detected in LOVO and SW480 cells co-transfected with WT-NOTCH1-3′-UTR or MUT-NOTCH1-3′-UTR and miRNA NC or miR-665 mimic (*n* = 3). (d) NOTCH1 mRNA level was examined in CRC tissues and adjacent normal tissues (*n* = 30). (e) NOTCH1 protein level was measured in NCM460, LOVO, and SW480 cells (*n* = 3). (f) NOTCH1 protein expression was determined in LOVO and SW480 cells transfected with pc-NC or pc-NOTCH1 (*n* = 3). (g) NOTCH1 protein level was detected in LOVO and SW480 cells transfected with miRNA NC, miR-665 mimic, miR-665 mimic + pc-NC, or miR-665 mimic + pc-NOTCH1 (*n* = 3). (h) NOTCH1 protein level was measured in LOVO and SW480 cells transfected with si-NC, si-circ_0005615, si-circ_0005615 + inhibitor NC, or si-circ_0005615 + miR-665 inhibitor (*n* = 3). (i) NOTCH1 protein level was detected in LOVO and SW480 cells treated with 6 Gy irradiation (*n* = 3). Data are shown as mean ± SD. *P < 0.05.

TargetScan prediction software showed that miR-665 and NOTCH1 3′-UTR had an assumed binding site (Figure 5a). Next, dual-luciferase reporter assay suggested that miR-665 mimic significantly decreased the luciferase activity of WT-NOTCH1 3′-UTR reporter (Figure 5b and c). As shown in Figure 5d, the NOTCH1 mRNA level in CRC tissues (1.51-fold) was markedly higher than that in normal tissues. Also, the NOTCH1 protein level in LOVO (1.96-fold) and SW480 (2.26-fold) cells was overtly increased in comparison with NCM460 cells (Figure 5e). Western blot assay exhibited that the overexpression efficiency of NOTCH1 was significant (Figure 5f). Moreover, the introduction of miR-665 mimic suppressed the expression of NOTCH1, while co-transfection of miR-665 mimic and pc-NOTCH1 mitigated the inhibitory effect (Figure 5g). Furthermore, inhibition of miR-665 reversed the decline in NOTCH1 protein level caused by circ_0005615 knockdown (Figure 5h). Besides, NOTCH1 protein expression was strikingly repressed in LOVO (with a 43% decrease) and SW480 cells (with a 54% reduction) exposed to 6 Gy irradiation (Figure 5i).

### 3.6 miR-665 mimic increases the radiosensitivity of CRC cells by targeting NOTCH1

**Figure 6:**
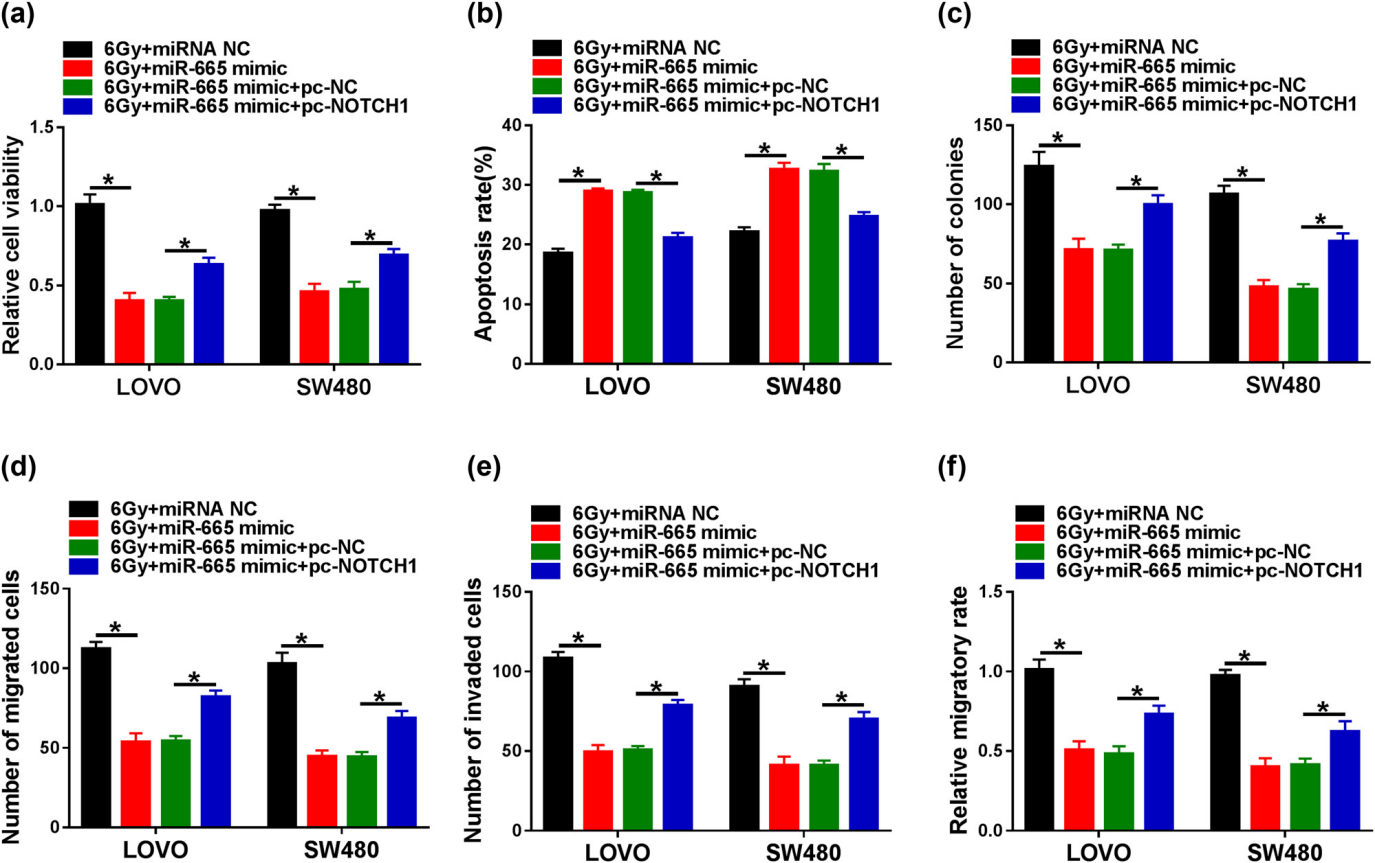
miR-665 mimic increases the radiosensitivity of CRC cells by targeting NOTCH1. LOVO and SW480 cells were introduced with miRNA NC, miR-665 mimic, miR-665 mimic + pc-NC, or miR-665 mimic + pc-NOTCH1 and then treated with 6 Gy irradiation. Cell viability (a), apoptosis (b), colony number (c), migration and invasion (d and e), and migratory rate (f) were detected using CCK-8 assay, flow cytometry, colony formation assay, transwell, and scratch assay, respectively. Data are shown as mean ± SD, *n* = 3. *P < 0.05.

To elucidate the association between miR-665 and NOTCH1 in the radiosensitivity of CRC cells, LOVO and SW480 cells transfected with miRNA NC, miR-665 mimic, miR-665 mimic + pc-NC, or miR-665 mimic + pc-NOTCH1 were treated with 6 Gy radiation. First, CCK-8 and flow cytometry assays revealed that miR-665 overexpression repressed cell viability and triggered apoptosis in LOVO and SW480 cells, while these changes were abolished after transfection with pc-NOTCH1 (Figure 6a and b). In addition, up-regulation of NOTCH1 alleviated the inhibition of miR-665 mimic on the proliferation of LOVO and SW480 cells (Figure 6c). Furthermore, transwell and scratch assays suggested that NOTCH1 overexpression attenuated the inhibitory effect of miR-665 mimic on the migration and invasion of LOVO and SW480 cells (Figure 6d–f). Collectively, these data evidenced that overexpression of miR-665 enhanced the radiosensitivity of CRC cells by regulating NOTCH1.

### 3.7 circ_0005615 silencing increases radiosensitivity *in vivo*

**Figure 7:**
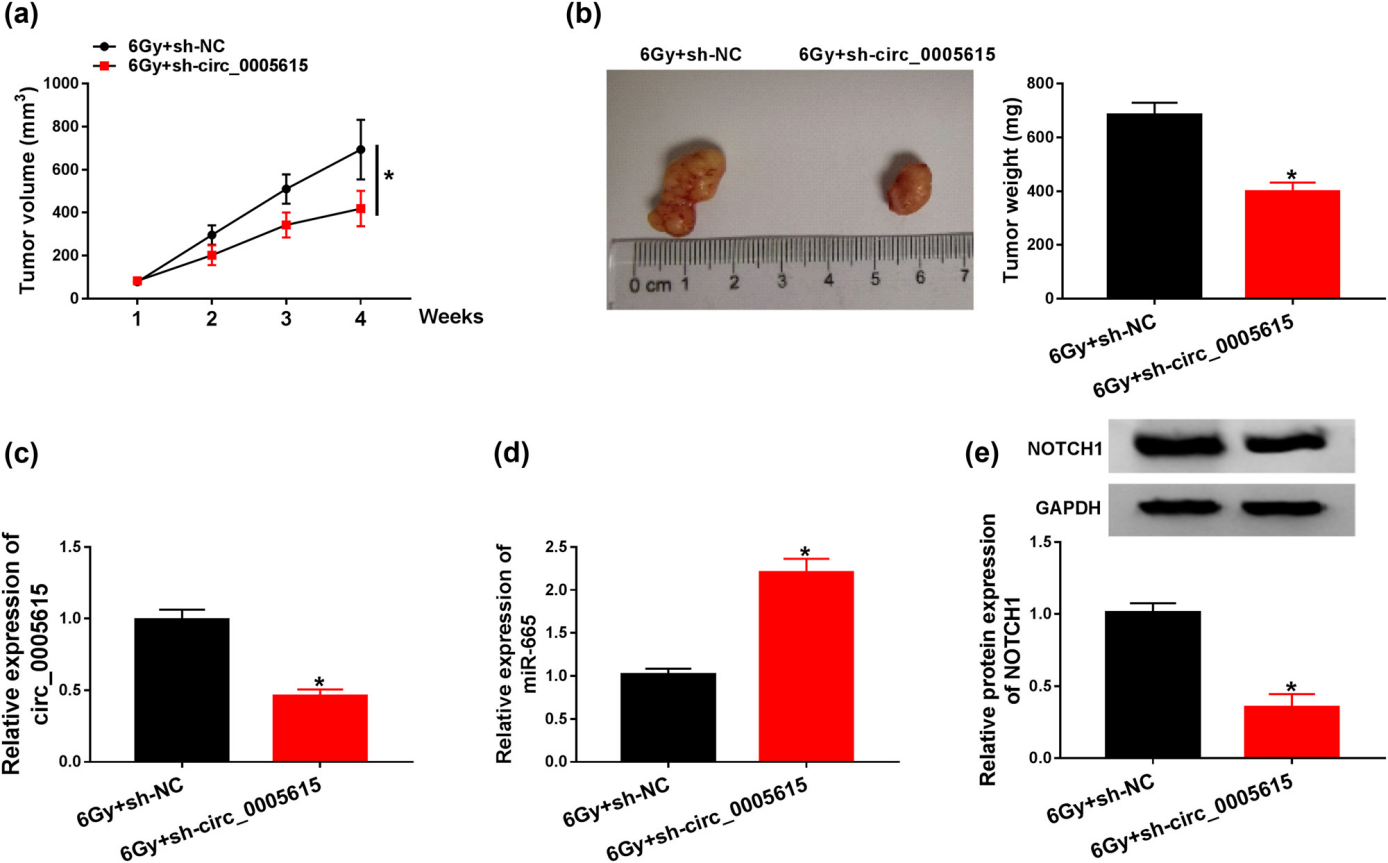
circ_0005615 silencing increases the radiosensitivity *in vivo*. Transfected LOVO cells with sh-circ_0005615 or sh-NC were subcutaneously injected into the nude mice. One week after injection, the tumors of the mice were exposed to 6 Gy radiation. (a) Tumor volume was measured once a week. (b) Mice were sacrificed 4 weeks later, and the tumors were removed and weighed. (c–e) Levels of circ_0005615, miR-665, and NOTCH1 in xenograft tumors were measured by qRT-PCR or western blot assay. Data are shown as mean ± SD, *n* = 5. *P < 0.05.

To explore the influence of circ_0005615 on radiosensitivity *in vivo*, we constructed a xenograft model. As illustrated in Figure 7a, tumor volume in the 6 Gy + sh-circ_0005615 group was markedly decreased compared to the 6 Gy + sh-NC group. Four weeks later, tumor weight in the 6 Gy + sh-circ_0005615 group was significantly reduced relative to the control group (Figure 7b). Additionally, silencing of circ_0005615 inhibited the expression of circ_0005615 and NOTCH1 and induced the expression of miR-665 (Figure 7c–e). These data indicated that circ_0005615 knockdown elevated radiosensitivity *in vivo*.

## 4 Discussion

Conventional surgical treatment combined with chemotherapy and radiotherapy has become the preferred strategy for the CRC [25]. Stereotactic body radiotherapy is a novel and effective therapy in the treatment of metastatic CRC [26]. Adjuvant radiotherapy reduces CRC recurrence rate and improves the survival rate [27]. Nonetheless, the emergence of radioresistance has become a huge obstacle to cancer therapy [28,29]. Radiation contributes to cancer cell death by regulating cell proliferation and apoptosis through the modulation of signaling pathways [30]. In-depth exploration of the molecular regulation of radioresistance is essential to improve the efficacy of radiotherapy. In recent years, some studies have manifested that not only lncRNAs and miRNAs, but also circRNAs are implicated in radiotherapy resistance of various cancers. It has been reported that dysregulated circ_0005615 was closely associated with the formation and development in different tumors [31,32], containing CRC [16]. However, the role and mechanism of circ_0005615 on radioresistance in CRC is still unclear. In the current research, our data first exhibited a competing endogenous RNAs (ceRNAs) network of circ_0005615/miR-665/NOTCH1 that was correlated with the radiosensitivity of CRC cells.

Here, we found that circ_0005615 content was obviously upregulated in CRC tissues and cell lines, consistent with a previous report [16]. Interestingly, our results identified high-level circ_0005615 might be associated with radioresistance in CRC for the first time, implying the positive effect of circ_0005615 in CRC. We inferred that the exact roles of circRNAs in cancers might be induced owing to the alteration of the tumor microenvironment. Under 6 Gy irradiation, circ_0005615 absence might improve radiosensitivity in CRC cells with the decrease in cell proliferation, migration, invasion, and increase in apoptosis. In addition, the radioresistence of circ_0005615 was verified on CRC xenografts in nude mice. These findings indicated the inimical function of circ_0005615 in radiotherapy for CRC patients.

In terms of mechanism, circRNAs regulate tumor progression by participating in many pathways, including serving as miRNA sponges [33]. For instance, circPITX1 silencing sensitized glioma to irradiation by declining glycolysis via competitively binding miR-329-3p to up-regulate NEK2 [34]. Liu et al. showed that circRNA_100367 strengthened the radioresistance of esophageal squamous cell carcinoma through functioning as a ceRNA for miR-217 and elevating Wnt3 [35]. Chen et al. disclosed that circRNA_000543 alleviated the radiosensitivity of nasopharyngeal carcinoma via sponging miR-9 and increasing PDGFRB expression [36]. In this research, we verified that circ_0005615 sponged miR-665 in CRC cells. Additionally, previous studies unveiled that miR-665 plays opposite roles in different cancers due to the differences in the tumor microenvironment. For example, miR-665 aggravated the malignancy of ovarian cancer via repressing SRCIN1 [37]. In gastric cancer, miR-665 hindered tumor growth and metastasis through down-regulating PPP2R2A [38]. In hepatocellular carcinoma, miR-665 facilitated cell proliferation and mobility via modulating PTPRB to inactivate Hippo signaling [39]. Hence, we selected miR-665 as the miRNA target of circ_0005615 for research. In the current research, rescue experiments suggested miR-665 knockdown abolished the effect of circ_0005615 silencing on CRC radiosensitivity.

Furthermore, substantial evidence has highlighted that miRNAs contribute to gene silencing via base-pairing with 3′-UTR of target genes [40]. Therefore, we searched the downstream target genes of miR-665 in the prediction database and identified NOTCH1 as the research object. Notch signaling exerts a crucial effect on tumorigenesis by regulating cell growth and development [41]. Revealing the interaction between Notch receptors and other pathways in CRC contributed to the exploration of the pathogenesis of CRC [42]. Besides, NOTCH1 is closely associated with radiation resistance in several cancers, including glioma [43], adenoid cystic carcinoma [44], and lung cancer [45]. Zhang et al. revealed that the depletion of NOTCH1 enhanced the radiosensitivity of CRC cells [46]. In this report, NOTCH1 up-regulation abrogated the effect of miR-665 mimic on CRC radiosensitivity.

**Figure 8:**
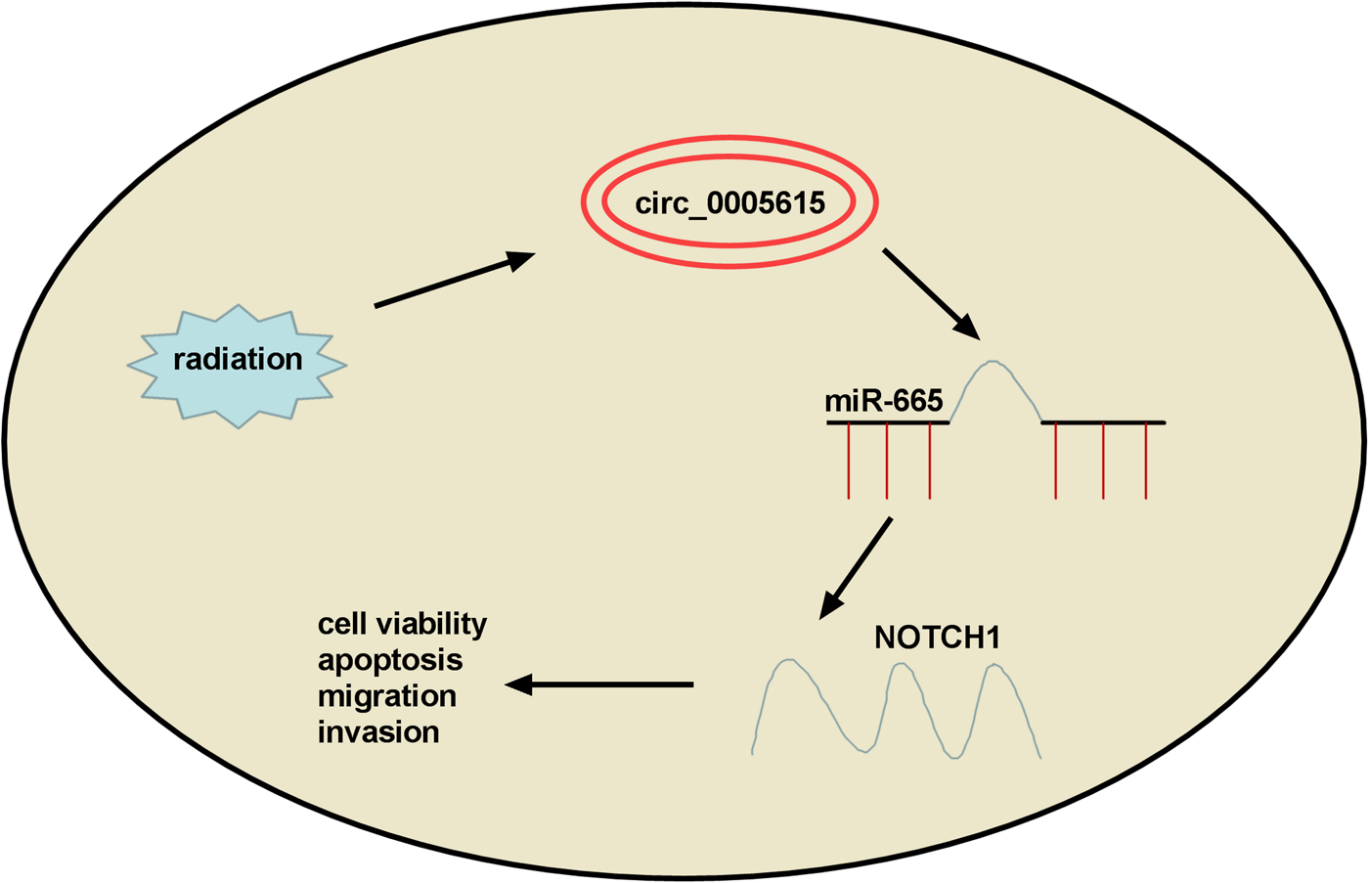
Schematic diagram of the regulation of circ_0005615/miR-665/NOTCH1 signaling pathway in colorectal cancer.

In conclusion, our findings unveiled that circ_0005615 elevated NOTCH1 expression by acting as a ceRNA for miR-665. Also, circ_0005615 strengthened the radioresistance of CRC via regulating miR-665/NOTCH1 signaling (Figure 8), suggesting that circ_0005615 might be a promising biomarker for CRC radiotherapy.
